# Œsophageal Obstruction.—III

**Published:** 1908-08-22

**Authors:** 


### SURGERY.

### ŒSOPHAGEAL OBSTRUCTION.—III.

### SOME REMARKS UPON ITS OPERATIVE TREATMENT.

The treatment of a simple stricture of the œsophagus is a comparatively easy matter, and is best effected by periodical dilatation of the canal by bougies under anæsthesia. These remarks are, therefore, confined to those cases in which the obstruction is definitely due to malignant disease.

The most optimistic surgeon is bound to confess, if he is an honest man, that the surgical treatment of malignant disease is, to say the least of it, highly unsatisfactory. A radical cure resulting from surgical interference in a case of carcinoma, even when this is situated in an exposed position, is the exception. And when the disease attacks an internal and deeply-seated viscus, the most that can generally be attempted is some form of palliative treatment directed to producing an artificial means for carrying on the functions of the affected organ.

This is the case in epithelioma of the œsophagus. It is true that attempts have been made to excise a malignant stricture in this situation, but none of them have been completely successful; that is to say, there is, as far as the author is aware, no recorded case of a patient who was still alive and well, say, one year after the operation.

The alternatives which face the surgeon are, therefore, (i.) to keep the œsophagus patent by means of a rigid tube passed through the growth, a method associated with the name of Mr. Symonds, or (ii.) to make an artificial opening into the stomach by means of the operation known as gastrostomy. Intubation with Symond's tube, ingenious as it is, has many disadvantages, and has been almost entirely superseded by gastrostomy. A short description of the method, however, will not be out of place. The tube consists of a catheter, four or five inches long, with a funnel-shaped expansion at its upper end and a terminal eye at its lower end. Long strings are attached to the margin of the funnel. The tube is introduced by the mouth with a long whalebone introducer, and lowered until it rests in the stricture. The funnel-shaped expansion rests on the growth and prevents the tube from passing through into the stomach. The strings are brought out of the mouth on either side and fixed to the ears or to the cheek with plaster. The advantages claimed for this method are that the stricture is kept open and the patient can still swallow his food naturally. But its disadvantages more than outweigh this. The presence of the strings which fix the tube in place is a source of constant irritation to the patient. Moreover, the tube gets very foul, and must be changed from time to time, and it has been argued that its presence irritates the growth and causes it to ulcerate and spread. Finally it has been known to slip through the growth into the stomach. This is not a very serious complication, because the tube becomes softened by digestive processes and eventually passes.

For these reasons gastrostomy has in recent years largely superseded intubation, so that in the treatment of a case of œsophageal obstruction the difficult point to decide is not what to do, but when to do it. Inasmuch as it is a palliative operation merely, it is obvious that there is no point in doing it as long as the patient can obtain sufficient nutrition *per vias naturales*. On the other hand, it must not be put off too long or the patient will become too debilitated to stand the shock of the operation. It is a sound rule to take the weight of the patient daily, or at any rate every other day, as soon as the nature of his trouble is known. As soon as he begins to lose weight appreciably the operation should at once be performed. *Per contra* one sometimes finds that a patient who first comes under observation with symptoms of the disease in an advanced form will show signs of improvement, and will even gain weight, if he is kept under observation in a hospital or a nursing home. This is partly due to the fact that he is systematically and properly fed. The apparent improvement will be even greater if he can take medicine by the mouth and a mixture containing belladonna and cocaine be given him; for it must be remembered that wherever one has to deal with obstruction to a hollow viscus in the body, even if the primary cause is carcinoma, the symptoms are due partly to the organic obstruction and partly to the secondary spasm. The belladonna and cocaine allay the spasm, and the patient is able to take more food than he has done for some time past. But again, it must be remembered that the improvement so produced is only temporary, and he will eventually go down hill again; and it can readily be known when this is beginning to occur if his weight is taken regularly. This generally happens at the end of a week or ten days, and that is the psychological moment at which gastrostomy should be done in such a case.

There are surgeons who say that gastrostomy is at the best of times an unjustifiable operation, and that it is better to let the patient die a natural death than to subject him to the inconvenience of an operation which will at the best only prolong his life for a short time. Only those who have suffered the pangs of a genuine starvation can appreciate the agony of it; and if one can believe the statements of patients in this condition, one must suppose that the horror of a gradual death from inanition is worse than that of undergoing a surgical operation. And then again this prejudice is partly based upon the want of success which attended the operation even a few years ago; but now that the technique of the operation has been improved the immediate result is rarely disastrous or even unsatisfactory, and the patient may live for some time in comparative comfort until he is eventually carried off by exhaustion or cachexia or the immediate results of the direct extension of carcinoma into the respiratory tract.

Several methods of performing the operation have been described, and these will be discussed in the succeeding article of this series.

